# Effectiveness and Tolerability of First-Line Afatinib for Advanced EGFR-Mutant Non-Small Cell Lung Cancer in Vietnam

**DOI:** 10.31557/APJCP.2021.22.5.1581

**Published:** 2021-05

**Authors:** Thanh Ha Vu, Hoa Thai Thi Nguyen, Linh Khanh Dao, Chi Khanh Duong, Cao Van Nguyen, Tuyet Thi Doan, Hang Thuy Thi Nguyen, Hung Huy Hoang, Dung Khac Dinh, Giang Vinh Le, Thanh Thi Vu, Minh Cong Truong, Long Thanh Nguyen

**Affiliations:** 1 *Department of Medical Oncology, Vietnam National Cancer Hospital, Hanoi, Vietnam. *; 2 *Department of Oncology, Vietnam University of Traditional Medicine, Hanoi, Vietnam. *; 3 *Department of Oncology, Hanoi Medical University, Hanoi, Vietnam.*

**Keywords:** Non small cell lung cancer (NSCLC), advanced stage, EGFR mutation, second generation EGFR TKI, real

## Abstract

**Background:**

We aimed to evaluate the effectiveness and tolerability of Afatinib as first-line treatment of advanced epidermal growth factor receptor (EGFR) mutant non small cell lung cancer (NSCLC) in a real-world setting.

**Patients and methods:**

This is a retrospective study of Vietnamese patients with advanced EGFR-mutant NSCLC treated with first-line afatinib at the National Cancer Hospital from 1st January 2018 to 31st October 2020. Patients’ demographic, clinical and treatment data were captured. Objective response rate (ORR), disease control rate (DCR), time to treatment failure (TTF) and tolerability were evaluated. We used Kaplan-Meier curve and log-rank test for survival, and Cox regression model for multivariate analysis.

**Results:**

A total of 44 patients were included. Common EGFR mutations (Del 19/L858R) were detected in 61% patients. Fifty percent of patients with uncommon mutations had compound mutations of G719X, L861Q and S768I. The ORR was 75% while DCR rate was 98%. The median TTF was 12.3 months (95% CI: 7.2-17.3); the mTTFs were 12.3 and 10.8 months for patients with common and uncommon mutations (p = 0.001), respectively, and 14.0 and 7.5 months for patients with Del 19 and L858R mutations (p = 0.067), respectively. Afatinib 30 mg once daily was the most common starting (77%) and maintenance (64%) doses. The mTTFs were 12.3 and 7.5 months for patients with 30 mg starting dose vs 40 mg dose (p = 0.256), respectively. Diarrhea, skin rash, paronychia and fatigue were observed in 32%, 30%, 25% and 9%, respectively. There was no grade 4 toxicity except three patients with grade 3 paronychia.

**Conclusions:**

First-line afatinib is beneficial for Vietnamese patients with advanced EGFR-mutant NSCLC with a good response rate and prolonged TTF with manageable adverse event profile. Baseline brain metastasis status and starting doses do not significantly impact TTF.

## Introduction

Lung cancer is the most common cause of cancer-related mortality worldwide. According to GLOBOCAN 2020, in Vietnam, lung cancer is a popular type of cancer with the second highest rate of mortality (Sung et al., 2021). The most common histologic pattern is adenocarcinoma, accounting for 40% of lung cancer cases, and squamous cell carcimona, with 20% of cases, is known as the next most prevalent subtype (Travis, 2011). On a report of recent local survey, about 80% cases of lung cancer in Vietnam presented as stage III/IV with widespread metastases (Thuan Tran Van, 2016). Advanced lung cancer has a low response rate to chemotherapy and an extremely poor prognosis (Planchard et al., 2018). From 2000s, management and treatment of lung cancer has transformed. This changed particularly when a small percentage of non small cell lung cancer (NSCLC) were identified with mutations in the EGFR gene that rendered those tumors sensitive to the EGFR tyrosine kinase inhibitors (TKI) (Hirsch et al., 2003; Mok et al., 2009; Rosell et al., 2012; Sequist et al., 2013; Wu et al., 2014; Wu et al., 2017; Planchard et al., 2018; Ramalingam et al., 2020). Afatinib, a second generation EGFR TKI which targets all members of ErbB family (Solca et al., 2012), proved the high efficacy in the first-line treatment of advanced NSCLC with EGFR mutations in many randomized controlled trials (RCTs) including LUX-Lung 3, 6 and 7 (Sequist et al., 2013; Wu et al., 2014; Park et al., 2016). On the results of these studies, afatinib was reported to significantly improve objective response rate (ORR) (range: 57-70%) and progression-free survival (PFS) (range: 11.0–11.1 months), relative to chemotherapy and gefitinib. Afatinib also showed eficacy in tumors with major uncommon EGFR mutations (G719X, L861Q and S768I) with ORR of 60-71% and mPFS of 11.0 months (Yang et al., 2015a; Yang et al., 2020) while the first generation EGFR TKIs gefitinib and elortinib showed the poor results on G719X and L861Q with ORR of 20-51% and mPFS of 2.2-6.0 months (Wu et al., 2011; Watanabe et al., 2014) .

Although Asian patients were enrolled in the randomized controlled trials for afatinib, few Vietnamese patients were recruited (Sequist et al., 2013; Wu et al., 2014). In reality, even though afatinib has been widely used in clinical pratice since 2013 (Dungo and Keating, 2013), there is still a lack of local real-world data on the effectiveness and tolerability of this second generation EGFR TKI in Vietnamese patients. In real-world settings, confounding factors during clinical practice may influence the clinical outcomes. Herein, we conducted a retrospective real-world study to explore the effectiveness and tolerability of first-line afatinib in Vietnamese patients with advanced EGFR-mutant NSCLC.

## Materials and Methods

*Patients*

We retrospectively screened advanced NSCLC patients treated with afatinib at the Vietnam National Cancer Hospital from 1st Januray 2018 to 31st October 2020. The ethics committee of the National Cancer Hospital approved the study. Patients who met the following criteria were included: (i) histologically confirmed Stage IIIC/IV NSCLC; (ii) sensitive EGFR mutations; (iii) treatment naïve with TKIs or other therapies; (iv) aged ≥ 18 years; and (v) hepatic, renal, and hematologic parameters met treatment eligibility. The exclusion criteria were: (i) severe hepatic or renal impairment or serious comobidities prevented the TKI treatment; (ii) allergic to afatinib or any of its excipient; (iii) failure to comply with treatment regimen or switch to another TKI; and (iv) accompanying with other malignant tumors. Peptide nucleic acid-mediated polymerase chain reaction clamping (PNA-PCR clamping) or new generation sequencing (NGS) were used to determine EGFR mutations. Patients received 20 mg, 30 mg or 40 mg afatinib daily as a starting dose. Afatinib 40 mg once daily is the recommended starting dose. The starting dose was determined at the discretion of the clinicians according to patient age, body surface area, Eastern Cooperative OncologyGroup performance status (ECOG PS), and so forth. Afatinib was given until symptomatic disease progression or occurrence of intolerable adverse events. Dosage reduction and drug discontinuation were followed as the manufacturer’s instructions (Boehringer Ingelheim). As for patients treated with 20 mg or 30 mg starting dose, after a month, if the treatment had been well tolerated, dosage of afatinib could be maintained or escalated by 10 mg once daily with clinician’s judgment. 

*Outcomes and evaluation*

The primary outcomes were to evaluate objective response rate (ORR), disease control rate (DCR) and time to treatment failure (TTF). ORR is defined as the ratio of total patients who received afatinib to patients experiencing complete response (CR) or partial response (PR) after the first evaluation of tumor response according to Response Evaluation Criteria in Solid Tumors, version 1.1 (RECIST 1.1) (Eisenhauer et al., 2009). DCR is defined as the ratio of total patients who received afatinib to patients experiencing CR, PR or stable disease (SD) after the first evaluation of tumor response. TTF is defined as the time from the first dose of afatinib to tumor progression based on RECIST 1.1 and/or the patient has a clinical progression assessed by investigators. Secondary outcome was to evaluate tolerability. Adverse events (AEs) were recorded through the clinical examination checklist and laboratory tests.

Clinical data were extracted from patients’ medical records and supplemented by follow-up if needed. Demographic and clinical data was collected. Patient performance status (PS) was assessed according to Eastern Cooperative Oncology Group (ECOG) score. Response to afatinib was evaluated by regular imaging examinations, in accordance with RECIST 1.1. Survival outcomes were collected from the initiation of afatinib treatment to the patient’s death or the end of the study on 31 October 2020. Adverse events were assessed using the National Cancer Institute Common Terminology Criteria for Adverse Events version 4.0 (CTCAE 4.0) .

*Statistical analysis*

Categorical variables were expressed as percentages and continuous variables were expressed as mean ± standard deviation (SD) or median with range depending on the normality of distribution of the variables. TTF was estimated using Kaplan-Meier method and shown as the estimated median time (months) and 95% confidence inderval (CI), respectively. Differences between categorical variables were tested using Chi-Squared test or Fisher’s Exact test. For continuous variables, the differences were compared using independent t-test or Mann-Whitney U test. All statistical analyses were tested independently and a statistically significant threshold was defined with p value of <0.05. Data was coded, inputed, and analysed using SPSS version 20.0 version (IBM Corp., Armonk, New York). 

## Results

*Patient characteristics and afatinib dose*

**Figure 1:**
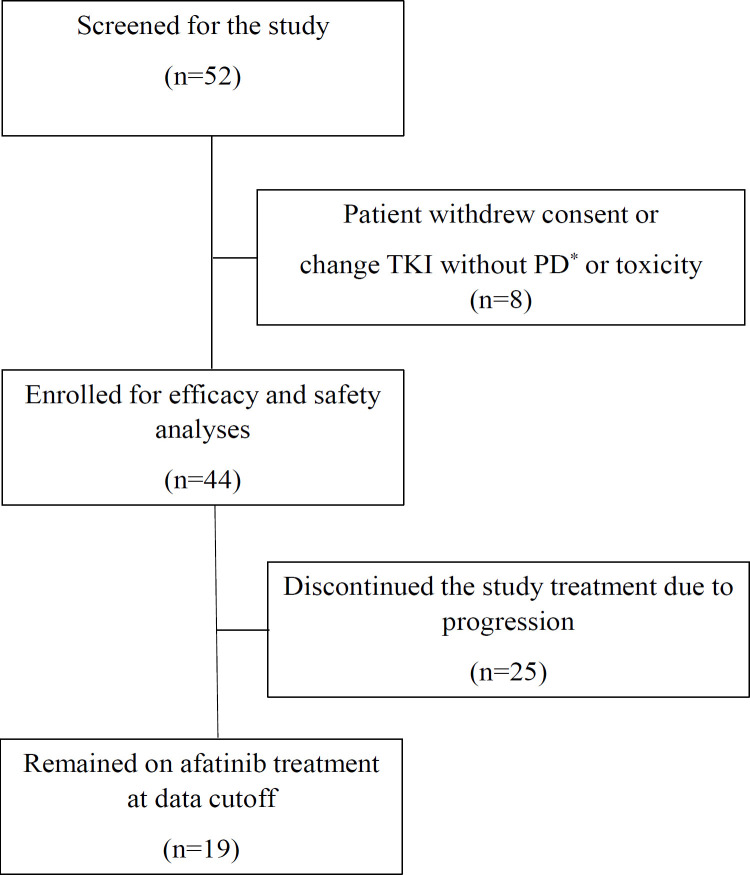
Study Algorithm

A total of 44 patients who met the study criteria were included (Figure 1). The clinical characteristics of the patients were shown in Table 1. All patients had lung adenocarcinoma with the mean age of 58 years (range: 32-76). Among them, the majority were male and ex-smoker or current smokers (61% and 59%, respectively). The ECOG performance status was 0-1 in 98%, symptomatic baseline brain metastases were present in 21% of the patients. The EGFR mutations included common mutations (Del 19/L858R) in 61%, Del 19 in 46%, L858R in 18%, and uncommon mutations in 36% of the patients. Among the uncommon mutations, the majority were G719X, L861Q and S768I with 50% in a compound form. One each had T263P, exon 20 insersion, and a very rare compound mutation of A750P+Del 19, respectively. Nine patients (21%) had a syptomatatic brain metastasis. Among them, 44% were treated with gamma knife (3 patients) or whole brain radiation (1 patient). 

Most of the patients started with afatinib 30 mg once daily (79%), followed by 40 mg once daily (16%) and 20 mg once daily (5%) (Table 2). Most of them could be maintained with the starting dose (63%) and the dose adjustments were required in 36%. Fourteen patients (32%) had dose increased based on their well-tolerated treatment after the first month and 2 patients (5%) had dose reduced due to adverse events. The optimal dose of afatinib was 30 mg once daily (57%), followed by 40 mg once daily (37%) and 20 mg once daily (5%).

*Effectiveness of afatinib treatment*

The results of ORR and DCR are shown in Table 3. At the first assessment (after 8 to 12 weeks), the complete response rate was 7%, patial response rate was 68%, and 10 patients achieved stable disease counting for 23%. The ORR was thus 75% while DCR was 98% on first-line afatinib. By 31st October 2020, 25 patients (57%) had disease progression with 4 (9%) with new brain lesions and 21 (48%) with new lesions at other sites. 

The subgrpup ORRs and DCRs are shown in Table 4. The patients with common and uncommon mutations had the similar ORRs (81.5% and 76.5%), and the same with Del 10 and L858R mutations (78.9% and 87.5%) or with 30 mg and 40 mg starting doses (80.0% and 71.4%). The similar ORRs were also observed in patients with dose increased and starting dose maintained (85.7% and 78.6%) or with optimal doses of 30 mg and 40 mg once daily (84.0% and 82.4%). No significant differences in ORR and DCR were observed on the univariate and multivariate analyses by EGFR mutation types, baseline brain metastasis status, afatinib starting doses, dose adjustments, and different optimal doses. 

**Figure 2:**
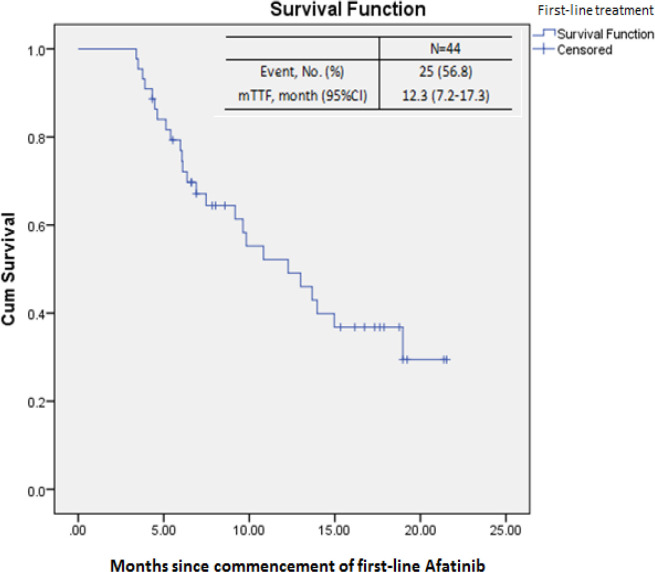
Kaplan-Meir Plot for Time to Treatment Failure of Patients on first-line Afatinib

Median TTF of the study population was 12.3 months (95% CI, 7.2–17.3) with 57% events (Figure 2). The mTTF according to EGFR mutation types, baseline brain metastases, staging, afatinib optimal doses and dose adjustments are shown in Table 5. On the univariate analysis, patients with common mutations (Del 19 and L858R) had significantly longer mTTF compared to rare EGFR mutations (12.3 vs 10.8 months; p = 0.001). No significant differences were observed in mTTF between patients with Del 19 and L858R mutations (14.0 vs 7.5 months, p = 0.067), and either with and without baseline brain metasteses (9.6 vs 13.0 months, p = 0.413), or with 30 mg and 40 mg starting doses (12.3 vs 7.5 months, p = 0.256). There were also no differences in mTTF between patients with dose increased and starting dose maintained (13.9 vs 8.9 months, p = 0.930), and the same with optimal doses of 30 mg and 40 mg once daily (9.8 vs 13.9 months, p = 0.256). The multivariate analysis did not further indicate any significant influence to the subgroup TTFs. 

*Tolerability of afatinib treatment*

Adverse event profile of afatinib was in line with expectations (Table 6). The most common adverse events (AEs) was diarrhea (32%). Skin rash was the second most common AE (30%). The incidence of paronychia, fatigue and liver enzyme elevation were 25%, 9% and 7%, respectively. There was no grade 4 AE. Only three patient with grade 3 paronycia (7%) required dose interruption. No patient required drug discontinuation or was exlcuded because of unacceptable toxicity. Though no significant differences were observed, the incidence of all grade diahrrea (67% vs 26%), skin rash (50% vs 29%), paronychia (33% vs 26%), and fatigue (33% vs 6%) was higher in patients with 40 mg starting dose than those with 30 mg dose. One patient (17%) with 40 mg starting dose experienced the grade 3 paronychia while two (6%) with 30 mg dose had.

**Table 1:** Baseline Characteristics of Patients with EGFR-Mutant NSCLC Treated with First-Line Afatinib during the Study Period

Characteristic	Patients (N=44)
Median age, years (range)	58 (32-76)
Gender, No. (%)	
Men	27 (61)
Women	17 (39)
ECOG performance status, No. (%)	
0	24 (55)
1	19 (43)
2	1 (2)
Smoking status, No. (%)	
Nerver Smoked	18 (41)
Ex/Current Smoked	26 (59)
Histology, No. (%)	
Adenocarcinoma	44 (100)
EGFR mutation, No. (%)	
G719X	5 (11)
G719X	2
G719X + S768I	3
G719C	2 (4)
G719C + L861Q	1
G719C + S786I	1
G719S + S768I	2 (4)
L861Q	2 (4)
L861Q	1
L861Q + G719A	1
S768I	7 (16)
S768I	3
S768I + G719C	1
S768I + G719X	3
S768I + G719S	2
L858R	8 (18)
T263P	1 (2)
Del19	20 (46)
Del19	19
Del19+ A750P	1
Exon 20 insertion	1 (2)
Brain Metastasis, No. (%)	9 (21)
Gamma knife	3
Whole brain Radiation	1
Overall disease classification, No. (%)	
IIIC	4 (9)
IVA	5 (11)
IVB	33 (75)
Recurrent	2 (5)

**Table 2:** Afatinib Starting Dose, Dose Adjustment and Optimal Dose

Dose modification	Patients (N=44)
Starting dose, No. (%)	
20 mg once daily	2 (5)
30 mg once daily	35 (79)
40 mg once daily	7 (16)
Afatinib dose adjustment, No. (%)	
Starting dose maintained	28 (63)
Dose increased	14 (32)
Dose reduced	2 (5)
Afatinib optimum dose, No. (%)	
20 mg once daily	2 (5)
30 mg once daily	25 (57)
40 mg once daily	17 (37)

**Table 3:** Treatment Outcome to Afatinib after 8-12 Weeks

Outcomes	Number of patients (n=44)
Response, No. (%)	
Complete response	3 (7)
Partial response	30 (68)
Stable disease	10 (23)
Progressive disease	1 (2)
Disease progression site, No. (%)	
None	19 (43)
New brain lesions	4 (9)
New lesions at other sites	21 (48)

*Following up by 31^st^ October 2020.

**Table 4:** Univariate and Multivariate Analyses of ORR and DCR According to Clinical and Treatment Characteristics

Characteristics	ORR, No. (%)	*p-value	^g^OR (95% CI), p-value	DCR	*p-value	^g^OR (95% CI), p-value
EGFR mutation subtype						
Del 19 (n=19)	15 (78.9)	0.222	0.255 (-0.8-0.45), 0.773^a^	19 (100)	0.427	0.063 (-0.15-0.15), 1.0^a^
L858R (n=8)	7 (87.5)		0.264 (-0.83-0.45), 0.759^b^	8 (100)		0.065 (-0.22-0.1), 0.609^b^
Uncommon (n=17)	13 (76.5)			16 (94.1)		
Common (n=27)	22 (81.5)			27 (100)		
Brain metastases						
Yes (n=9)	7 (77.8)	0.128	0.234 (-0.475-0.469), 0.128	8 (89)	0.077	0.111 (-0.145- 0.367), 0.077
No (n=35)	31 (88.6)			35 (100)		
Afatinib starting dose						
20 mg once daily (n=2)	2 (100)	0.961	0.46 (-1-1.23), 0.967^c^	2 (100)	0.884	0.112 (-0.24-0.3), 0.965^c^
30 mg once daily (n=35)	28 (80.0)		0.262 (-0.61-0.67), 0.993^d^	34 (97.1)		0.064(-0.18-0.13), 0.895^d^
40 mg once daily (n=7)	5 (71.4)			7 (100)		
Afatinib dose adjustment						
Dose reduced (n=2)	1 (50)e	0.718	0.158(-0.38-0.38), 1.0^f^	2 (100)	0.351	0.049 (-0.19-0.05), 0.325^f^
Dose increased (n=14)	12 (85.7)			13 (92.9)		
Starting dose maintained (n=28)	22 (78.6)			28 (100)		
Optimal afatinib dose						
20 mg once daily (n=2)	1 (50.0)	0.253	0.152 (-0.4-0.33), 0.971	2 (100)	0.462	0.048 (-0.06-0.17), 0.440
30 mg once daily (n=25)	21 (84.0)			25 (100)		
40 mg once daily (n=17)	14 (82.4)			16 (94.1)		

ORR, objective response rate; DCR, disease control rate; OR, odd ratio; 95% CI, 95% confidence interval; EGFR, epidermal growth factor receptor; ECOG, Eastern Cooperative Oncology Group; *p-value of Chi-squared test; ^a^Del 19 vs L858R; ^b^Del 19 vs rare mutations; ^c^afatinib dose 30 mg once daily 20 mg once daily; ^d^afatinib dose 40 mg once daily vs 30 mg once daily; ^e^not compared because of the small number of patients; ^f^afatinib dose increased versus starting dose maintained; gmultivariate analysis with cox regression.

**Table 5.** Analyses of TTF According to Clinical and Treatment Characteristics

Characteristics	Patients,	mTTF (months)	Univariate analysis, p-value*	Multivariate analysis^g^	
	No. (%)			HR	p-value*
EGFR mutation subtype					
Del 19	19 (43)	13.97	0.181	0.528 (0.170-1.646)	0.221
L858R	8 (18)	7.47	0.067^a^	1.323 (0.362-4.853)	
Rare + compound	17 (39)	10.83	0.236^b^		
Common (Del19/L858)	27 (61)	12.27	0.426^c^		
			0.001^d^		
Baseline brain metastasis					
Yes	9 (21)	9,6	0.413	0.957 (0.258-3.549)	0.948
No	35 (79)	13			
Afatinib starting dose					
20 mg once daily	2 (5)	Not reach	0.256	0.198 (0.054-7.402)	0.087
30 mg once daily	35 (79)	12.3			
40 mg once daily	7 (16)	7.5			
Afatinib dose adjustment					
Dose reduced	2 (5)	3.5	0.930^e^	0.767 (0.233-2.526)	0.768
Dose increased	14 (32)	13.9		1.832 (0.209-16.036)	0.988
Starting dose maintained	28 (63)	9.8			
Optimal afatinib dose					
20 mg once daily	2 (5)	3.5	0.448	0.634 (0.054-7.402)	0.546
30 mg once daily	25 (57)	9.8	0.174^f^	0.547 (0.189-1.713)	0.271^f^
40 mg once daily	17 (38)	13.6			

mTTF, median time to treatment failure; EGFR epidermal growth factor receptor; *p-value of Chi-square test; ^a^Del 19 vs L858R; ^b^Del 19 vs rare mutations; ^c^Rare mutations v L858R; ^d^common vs rare mutations; ^e^afatinib dose increased vs starting dose maintained; ^f^afatinib 40 mg once daily vs 30 mg once daily; ^g^multivariate analysis with cox regression.

**Table 6:** Treatment-Related Adverse Events Recorded in at Least 5% of Patients

Adverse event	All patients* N=44		30 mg OD n=35		40 mg OD n=6		p-value
	No.	%	No.	%	No.	%	
Diarrhea	14	32	9	26	4	67	0.108
≥ Grade 3	0	0	0	0	0	0	-
Rash	13	30	10	29	3	50	0.677
≥ Grade 3	0	0	0	0	0	0	-
Paronychia	11	25	9	26	2	33	0.997
≥ Grade 3	3	7	2	6	1	17	0.656
Fatigue	4	9	2	6	2	33	0.746
≥ Grade 3	0	0	0	0	0	0	-
Increased ALT/AST	3	7	3	9	0	0	0.705
≥ Grade 3	0	0	0	0	0	0	-
Not assessable	2	5	-	-	-	-	-

*There was no grade 4 adverse event and only one patient with 20 mg OD starting dose had grade 2 diarrhea (50%).

## Discussion

This study is the first retrospective, real-world study of the effectiveness and tolerability of first-line afatinib in Vietnamese patients with advanced EGFR-mutant NSCLC. In this study, all patients were adenocarcinoma with sensitive EGFR mutations, 98% had a relatively good ECOG PS score of 0-1 and the median age of all patients was 58 years, which made the results of this study comparable to LUX-Lung 3 and 6 trials (Sequist et al., 2013; Wu et al., 2014) .

The randomized control trials (RCTs) of LUX-Lung 3 and 6 reported the ORR, DCR and mPFS of first-line afatinib treatment of 57-68%, 90-93% and 11.0–11.1 months, respectively (Sequist et al., 2013; Wu et al., 2014). The mPFS even reached 13.6 months in patients with common mutations (Del 19/L858R). In our study, the response evaluation after 8-12 months in 44 patients showed ORR of 75% and DCR of 98%. The median TTF was 12.3 months in all patients as well as in patients with common mutations. Our results were comparable to those reported in RCTs. Further, our study included 61% of patients with common mutations. Among them, 19 (49%) had Del 19 and 8 (18%) had L858R mutation. The proportion of patients with Del 19 and L858R mutations in our study were lower than that in RCTs. The subgroup analysis of PFS in RCTs shows that the HR of Del 19 was superior to that of L858R when compared to chemotherapy (Sequist et al., 2013; Wu et al., 2014). Further, the pooled analyses indicates that afatinib demonstrated a significant improvement in OS in Del 19 subgroup vs chemotherapy (Yang et al., 2015b). Our results indicate that patients with Del 19 had longer mTTF than those with L858R mutation (14.0 vs 7.5 months), which conresponds to those reported in RCTs. Several real-world studies have also demonstrated this tendency and reported longer mPFS in Del 19 groups (Yang et al., 2015b). Kim and colleagues revealed that, in a subgroup of patients with Del 19, the median PFS of afatinib was significantly longer to those of gefitinib or erlotinib (19.1 vs 15.0 and 16.3 months, respectively; p = 0.01). However, there was no such significant difference in L858R subgroup (p = 0.46) (Kim et al., 2019). These outcomes may lead to a tendency in clinical practice to prescribe afatinib to patients with Del 19 disease.

The combined post-hoc analysis of LUX-Lung 2, 3 and 6 trials indicates that afatinib is active in patients with uncommon EGFR mutations (Yang et al., 2015a). Patients with major uncommon mutations (G719X, L861Q and S768I) with first-line afatinib treatment could achieve a median PFS of 10.7 months (95% CI 5.6-14.7). This is further supported by a pooled database analysis, where afatinib demonstrated activity against major uncommon mutations (mTTF = 10.8 months; 95% CI: 8.1-16.6; ORR = 60.0%) and compound mutations (mTTF = 14.7 months; 95% CI: 6.8-18.5; ORR = 77.1%) in 315 EGFR TKI-naïve patients (Yang et al., 2020). Interestingly, the first generation EGFR TKIs showed a poor effectiveness in uncommon mutations. For example, in NEJ002 study (Watanabe et al., 2014), the patients with G719X and L861Q mutations in gefitinib arm in first-line setting only achieved ORR of 20% and mPFS of 2.2 months. Wu and colleagues also showed ORR of 51% and mPFS of 6.0 months in patients with G719 and L861 mutations treated with first-line gefitinib or erlortinib (Watanabe et al., 2014). Similarly, in a phase II study of osimertinib in 36 patients with uncommon mutations with 61% in first-line treatment, patients with G719X, L861Q, S768I and their compound mutations achieved ORR of 50% and mPFS of 8.2 months (Cho et al., 2020). In our study, 36% of patients were with uncommon mutations. Among them, the majority were G719X, L861Q and S768I with 50% in a compound form, and one each had T263P, exon 20 insersion, and a very rare compound mutation of A750P+Del 19, respectively. Even though, our results showed that patients with uncommon mutations could achieved ORR of 77% and mTTF of 10.8 months in first-line afatinib treatment. This is consistent with those reported from the combined and pooled analyses on afatinib clinical activity in NSCLC with uncommon mutations (Yang et al., 2015a; Yang et al., 2020). Other real-world studies have also reported such clinical activity of afatinib and even found that patients with uncommon mutatoins had longer or significantly longer mPFS with afatinib therapy than those with first generation EGFR TKIs (Shen et al., 2017; Kim et al., 2019; Tanaka et al., 2019). 

Another combined analysis of LUX-Lung 3 and 6 trials demonstrates that afatinib provided a significant improvement in ORR and PFS in patients with brain metastases and common mutations compared to chemotherapy (73 vs 24%; p < 0.01; and 8.2 vs 5.4 months; HR 0.50; p = 0.0297, respectively) (Schuler et al., 2016). The extent of improvement in PFS from afatinib treatment of these patients was similar to that of patients without brain metastases, suggesting that afatinib represents a viable treatment option for the lesions in central nurvous system (CNS). The median cerebrospinal fluid penetration rate for afatinib of 2.45±2.91% has also been reported (Tamiya et al., 2017), confirming afatinib’s blood-barrier penetration and CNS activity. In our study, the ORR in patients with and without baseline brain metastases were 78% and 89%, respectively. Only 4 (9%) patients with disease progression developed new brain lesions. Further, the mTTF in patients with and without baseline brain metastases were 9.6 vs 13.0 months, respectively. The multivariate Cox regression analysis confirmed no significant difference in ORR and TTF between patients with and without baseline brain metastases. These results were satisfactory relative to the combined analysis (Schuler et al., 2016) though we recorded a limited number with only nine patients with brain metastases. Several real-world studies have also reported afatinib CNS activity with mPFS of 5.2-13.5 months (Liang et al., 2017; Tan et al., 2018; Ho et al., 2019). Tan and colleagues noticed that, in patients with brain metastases who treated with afatinib 40 mg starting dose was associated with improved PFS compared to 30 mg dose (13.3 vs 5.2 months; p = 0.006) (Tan et al., 2018). It is not clear how patients with 30 mg starting dose were monitored and managed during the treatment in their center. In our study, the patients received 20 mg or 30 mg starting dose were monitored, and the dosage of afatinib was maintained or escalated by 10 mg once daily based on their tolerability in the first month treatment. Most of the patients started with afatinib 30 mg once daily (79%), required no dose adjustment (63%), and stayed at the optimal dose of 30 mg once daily (57%) at the end. Our satisfactory results in ORR and TTF with a low CNS progression rate in a small number of patients with brain metasteses indicate the importance of dose escalation for some patients in control of CNS disease as the benefit of dose of afatinib on CNS disease is driven by the peak plasma concentrations attained (Yap et al., 2010). The afatinib CNS activity has been further supported in the real-world multi-cohort study with 559 patients by Jung and colleagues (Jung et al., 2020), where afatinb demonstrated 73% of CNS response rate and supior median CNS-PFS than gefitinib and erlotinib (23.3 vs 17.3 and 12.4 months, respectively; p < 0.001). On the competing risk analysis for cumulative incidence of CNS failure, afatinib further showed CNS prevention with a lower cumulative incidence of CNS failure compared with gefitinib or erlotinib after adjusting for both EGFR mutation types and preexisting CNS metastases (HR 0.51; 95% CI: 0.34–0.75; p = 0.0007). Currently, only FLAURA trial indicates that osimertinib had better CNS activity and prevention than gefitinib or erlotinib (median CNS-PFS: not reached vs 13.9 months; HR 0.48; 95% CI: 0.26-0.86; p = 0.014; and a lower probability of experiencing a CNS progression event with osimertinib vs gefitinib or erlotinib, respectivly) (Reungwetwattana et al., 2018). It seems that afatinib and osimertinib have a similar profile in CNS activity and prevention. 

Afatinib 40 mg once daily is the recommended starting dose. Tolerability-guided dose adjustment as recommended by the manufacturer has been demonstrated as an effective measure to reduce afatinib-related AEs without affecting therapeutic efficacy in LUX-Lung 3, 6 and 7 trials (Yang et al., 2016; Schuler et al., 2019). Dose reduction was more likely in patients with higher afatinib plasma concentrations and, on day 43, patients with dose reduction from 40 mg to 30 mg had geometric mean afatinib plasma concentrations of 23.3 ng/ml vs 22.8 ng/ml in patients who remained on 40 mg (Yang et al., 2016). The median PFS was similar in patients with dose reduction during the first 6 months vs those who did not (LUX-Lung 3: 11.3 versus 11.0 months, HR 1.25; LUX-Lung 6: 12.3 vs 11.0 months, HR 1.00; LUX-Lung 7: 12.8 vs 11.0 months, HR 1.34) (Yang et al., 2016; Schuler et al., 2019). These results in RCTs have been further supported by several real-world studies (Liang et al., 2017; Liu et al., 2017; Lim et al., 2018; Halmos et al., 2019; Ho et al., 2019; Tanaka et al., 2019; Igawa et al., 2020). Yang et al., (2017) further revealed in a real-world study that afatinib 30 mg starting dose had similar ORR and mPFS as the 40 mg dose (76 vs 95%, p = 0.0862; and 15.4 vs 14.6 months, p = 0.8418, respectively), but resulted in fewer serious drug-related AEs. Halmos et al., (2019) further demonstrated in the international real-world study with 228 EGFR-mutant NSCLC patients that there was no significant difference in TTF (p = 0.543) between patients who remained on afatinib ≥ 40 mg/day for the first 6 months (19.5 months; 95% CI: 13.4–not evaluable (Cho et al., 2020), who reduced dose to < 40 mg/day within the first 6 months (17.7 months; 95% CI: 14.5–21.5), and who started with afatinib ≤ 30 mg/day (19.4 months; 95% CI: 12.9-NE). In our study, most of patients started with afatinib 30 mg once daily (79%), followed by 40 mg once daily (16%) and 20 mg once daily (5%). This reflects our practice which may be different from other real-world studies (Liang et al., 2017; Liu et al., 2017; Yang et al., 2017; Lim et al., 2018; Halmos et al., 2019; Ho et al., 2019; Tanaka et al., 2019) . In our center, the clinicians usually started with a dose lower than standard dose to probe tolerance of patients based on their age, body surface area, ECOG PS and others as well as the previous real-word evidence (Yang et al., 2017; Halmos et al., 2019). After monitoring for a month, if the treatment had been well tolerated, dosage of afatinib could be maintained or escalated by 10 mg once daily accordingly. Our results indicated that patients with different starting dose of 20 mg, 30 mg and 40 mg had ORR of 100%, 80% and 71%, respectively. The mTTF was not reached in patients with 20 mg starting dose (small number) while patients with 30 mg or 40 mg starting dose had mTTF of 12.3 months and 7.5 months, respectively. No significant differences were found in ORR and TTF by the multivariate analysis. On the other hand, most of the patients could maintain with the starting dose (63%) and the dose adjustments were required in 36%. The subgroup of patients without afatinib dose adjustment had mTTF of 9.8 months while patients with dose increment reached mTTF of 13.9 months (p = 0.930). The mTTF in patients with the optimal dose of 40 mg once daily was higher than those with 30 mg and 20 mg once daily (13.9 vs 9.8 and 3.5 months, respectively) (p > 0.05). Furthermore, our practice also generated a well tolerable AE profile of afatinib in Vietnamese patients. The most common AE was diarrhea (32%), followed by skin rash (30%), paronychia (25%), fatigue (9%) and liver enzyme elevation (7%), respectively. There was no grade 4 AE. Only three patients with grade 3 paronycia required dose interruption. Patients who treated with 40 mg starting dose were more likely to experience diarrhea, skin rash, paronychia and fatigue than those with 30 mg starting dose. The incidence of grade 3 paronychia was more than double in patients with 40 mg starting dose vs 30 mg dose (17% vs 6%). Our results were similar to those of other real-world studies (Liang et al., 2017; Liu et al., 2017; Yang et al., 2017; Lim et al., 2018; Kim et al., 2019; Tanaka et al., 2019; Igawa et al., 2020), reporting a much lower frequency of grade 3 or 4 afatinib-related AEs when compared to 36.0–57.0% in RCTs (Wu et al., 2014; Park et al., 2016; Wu et al., 2017). This could have been due to the fact that the clinicians have got increased familiarity with afatinib and more expeience in managing the drug-related AEs. Our practice with a lower starting dose to probe patient’s tolerability followed by monitoring for dose maintainance or escalation subsequently could keep the optimal dose with less toxicity for patients in controlling the disease. This seems to work well with Vietnamese patients and is reassuring to the clinicians.

There were some limitations in the current study. Firstly, the retrospective nature and small sample size of the study may have introduced unavoidable bias. Secondly, the number of patients with L858R and uncommon mutations were disproportionally low and high, respectively. Thirdly, only 57% of the patients with disease progression were evaluated. A further study with a larger sample size is needed.

In conclusion, first-line afatinib treatment is effective in Vietnamese patients with advanced EGFR-mutant NSCLC. It is associated with a good response rate and prolonged TTF with well manageable AE profile. Baseline brain metastasis status and initial dose of afatinib do not significantly impact TTF. Our results warrant for further study with a larger sample size.

## Author Contribution Statement

Conception and design: Vu Ha Thanh, Nguyen Thi Thai Hoa Administrative support: Vu Ha Thanh, Nguyen Thi Thai Hoa Collection and assembly of data: Vu Ha Thanh, Nguyen Thi Thai Hoa, Dao Khanh Linh, Duong Khanh Chi, Nguyen Van Cao, Doan Thi Tuyet, Nguyen Thi Thuy Hang, Le Vinh Giang, Hoang Huy Hung, Dinh Khac Dung, Vu Thi Thanh, Truong Cong Minh, Nguyen Thanh Long Data analysis and interpretation: Vu Ha Thanh, Nguyen Thi Thai Hoa, Truong Cong Minh, Nguyen Thanh Long, Manuscript writing: All authors Final approval of manuscript: All authors Accountable for all aspects of the work: All authors.

## References

[B1] Cho JH, Lim SH, An HJ (2020). Osimertinib for patients with non-small-cell lung cancer harboring uncommon EGFR mutations: A Multicenter, Open-Label, Phase II Trial (KCSG-LU15-09). J Clin Oncol.

[B2] Dungo RT, Keating GM (2013). Afatinib: first global approval. Drugs.

[B3] Eisenhauer EA, Therasse P, Bogaerts J (2009). New response evaluation criteria in solid tumours: revised RECIST guideline (version 1. Eur J Cancer.

[B4] Halmos B, Tan EH, Soo RA (2019). Impact of afatinib dose modification on safety and effectiveness in patients with EGFR mutation-positive advanced NSCLC: Results from a global real-world study (RealGiDo). Lung Cancer.

[B5] Hirsch FR, Varella-Garcia M, Bunn PA, Jr. (2003). Epidermal growth factor receptor in non-small-cell lung carcinomas: correlation between gene copy number and protein expression and impact on prognosis. J Clin Oncol.

[B6] Ho GF, Chai CS, Alip A (2019). Real-world experience of first-line afatinib in patients with EGFR-mutant advanced NSCLC: a multicenter observational study. BMC Cancer.

[B7] Igawa S, Ono T, Kasajima M (2020). Real-world assessment of afatinib for patients with EGFR-positive non-small cell lung cancer. Invest New Drugs.

[B8] Jung HA, Woo SY, Lee SH (2020). The different central nervous system efficacy among gefitinib, erlotinib and afatinib in patients with epidermal growth factor receptor mutation-positive non-small cell lung cancer. Transl Lung Cancer Res.

[B9] Kim Y, Lee SH, Ahn JS (2019). Efficacy and safety of afatinib for EGFR-mutant non-small cell lung cancer, compared with gefitinib or erlotinib. Cancer Res Treat.

[B10] Liang SK, Hsieh MS, Lee MR (2017). Real-world experience of afatinib as a first-line therapy for advanced EGFR mutation-positive lung adenocarcinoma. Oncotarget.

[B11] Lim CK, Wei YF, Tsai MS (2018). Treatment effectiveness and tolerability of afatinib at different doses in patients with EGFR-mutated lung adenocarcinoma: How low can we go?. Eur J Cancer.

[B12] Liu CY, Wang CL, Li SH (2017). The efficacy of 40 mg versus dose de-escalation to less than 40 mg of afatinib (Giotrif) as the first-line therapy for patients with primary lung adenocarcinoma harboring favorable epidermal growth factor mutations. Oncotarget.

[B13] Mok TS, Wu Y-L, Thongprasert S (2009). Gefitinib or carboplatin–paclitaxel in pulmonary adenocarcinoma. N Engl J Med.

[B14] Park K, Tan EH, O’Byrne K (2016). Afatinib versus gefitinib as first-line treatment of patients with EGFR mutation-positive non-small-cell lung cancer (LUX-Lung 7): a phase 2B, open-label, randomised controlled trial. Lancet Oncol.

[B15] Planchard D, Popat S, Kerr K (2018). Metastatic non-small cell lung cancer: ESMO Clinical Practice Guidelines for diagnosis, treatment and follow-up. Ann Oncol.

[B16] Ramalingam SS, Vansteenkiste J, Planchard D (2020). Overall survival with osimertinib in untreated, EGFR-mutated advanced NSCLC. N Engl J Med.

[B17] Reungwetwattana T, Nakagawa K, Cho BC (2018). CNS response to osimertinib versus standard epidermal growth factor receptor tyrosine kinase inhibitors in patients with untreated EGFR-mutated advanced non-small-cell lung cancer. J Clin Oncol.

[B18] Rosell R, Carcereny E, Gervais R (2012). Erlotinib versus standard chemotherapy as first-line treatment for European patients with advanced EGFR mutation-positive non-small-cell lung cancer (EURTAC): a multicentre, open-label, randomised phase 3 trial. Lancet Oncol.

[B19] Schuler M, Tan EH, O’Byrne K (2019). First-line afatinib vs gefitinib for patients with EGFR mutation-positive NSCLC (LUX-Lung 7): impact of afatinib dose adjustment and analysis of mode of initial progression for patients who continued treatment beyond progression. J Cancer Res Clin Oncol.

[B20] Schuler M, Wu YL, Hirsh V (2016). First-line afatinib versus chemotherapy in patients with non-small cell lung cancer and common epidermal growth factor receptor gene mutations and brain metastases. J Thorac Oncol.

[B21] Sequist LV, Yang JC, Yamamoto N (2013). Phase III study of afatinib or cisplatin plus pemetrexed in patients with metastatic lung adenocarcinoma with EGFR mutations. J Clin Oncol.

[B22] Shen YC, Tseng GC, Tu CY (2017). Comparing the effects of afatinib with gefitinib or Erlotinib in patients with advanced-stage lung adenocarcinoma harboring non-classical epidermal growth factor receptor mutations. Lung Cancer.

[B23] Solca F, Dahl G, Zoephel A (2012). Target binding properties and cellular activity of afatinib (BIBW 2992), an irreversible ErbB family blocker. J Pharmacol Exp Ther.

[B24] Sung H, Ferlay J, Siegel RL (2021). Global cancer statistics 2020: GLOBOCAN estimates of incidence and mortality worldwide for 36 cancers in 185 countries.

[B25] Tamiya A, Tamiya M, Nishihara T (2017). Cerebrospinal fluid penetration rate and efficacy of afatinib in patients with EGFR mutation-positive non-small cell lung cancer with leptomeningeal carcinomatosis: A Multicenter Prospective Study. Anticancer Res.

[B26] Tan W-L, Ng QS, Lim C (2018). Influence of afatinib dose on outcomes of advanced EGFR-mutant NSCLC patients with brain metastases. BMC Cancer.

[B27] Tanaka I, Morise M, Kodama Y (2019). Potential for afatinib as an optimal treatment for advanced non-small cell lung carcinoma in patients with uncommon EGFR mutations. Lung Cancer.

[B28] Thuan Tran Van APT, Tu Dao Van (2016). Cancer control in Vietnam: where are we now. Cancer Control.

[B29] Travis WD (2011). Pathology of lung cancer. Clin Chest Med.

[B30] Watanabe S, Minegishi Y, Yoshizawa H (2014). Effectiveness of gefitinib against non-small-cell lung cancer with the uncommon EGFR mutations G719X and L861Q. J Thorac Oncol.

[B31] Wu JY, Yu CJ, Chang YC (2011). Effectiveness of tyrosine kinase inhibitors on “uncommon” epidermal growth factor receptor mutations of unknown clinical significance in non-small cell lung cancer. Clin Cancer Res.

[B32] Wu Y-L, Cheng Y, Zhou X (2017). Dacomitinib versus gefitinib as first-line treatment for patients with <em>EGFR</em>-mutation-positive non-small-cell lung cancer (ARCHER 1050): a randomised, open-label, phase 3 trial. Lancet Oncol.

[B33] Wu YL, Zhou C, Hu CP (2014). Afatinib versus cisplatin plus gemcitabine for first-line treatment of Asian patients with advanced non-small-cell lung cancer harbouring EGFR mutations (LUX-Lung 6): an open-label, randomised phase 3 trial. Lancet Oncol.

[B34] Yang CJ, Tsai MJ, Hung JY (2017). The clinical efficacy of Afatinib 30 mg daily as starting dose may not be inferior to Afatinib 40 mg daily in patients with stage IV lung Adenocarcinoma harboring exon 19 or exon 21 mutations. BMC Pharmacol Toxicol.

[B35] Yang JC-H, Schuler M, Popat S (2020). Afatinib for the treatment of NSCLC harboring uncommon <em>EGFR</em> mutations: A Database of 693 Cases. J Thorac Oncol.

[B36] Yang JC, Sequist LV, Geater SL (2015a). Clinical activity of afatinib in patients with advanced non-small-cell lung cancer harbouring uncommon EGFR mutations: a combined post-hoc analysis of LUX-Lung 2, LUX-Lung 3, and LUX-Lung 6. Lancet Oncol.

[B37] Yang JC, Sequist LV, Zhou C (2016). Effect of dose adjustment on the safety and efficacy of afatinib for EGFR mutation-positive lung adenocarcinoma: post hoc analyses of the randomized LUX-Lung 3 and 6 trials. Ann Oncol.

[B38] Yang JC, Wu YL, Schuler M (2015b). Afatinib versus cisplatin-based chemotherapy for EGFR mutation-positive lung adenocarcinoma (LUX-Lung 3 and LUX-Lung 6): analysis of overall survival data from two randomised, phase 3 trials. Lancet Oncol.

[B39] Yap TA, Vidal L, Adam J (2010). Phase I trial of the irreversible EGFR and HER2 kinase inhibitor BIBW 2992 in patients with advanced solid tumors. J Clin Oncol.

